# Tools for faculty assessment of interdisciplinary competencies of healthcare students: an integrative review

**DOI:** 10.3389/fmed.2023.1124264

**Published:** 2023-06-16

**Authors:** Sharon Brownie, Denise Blanchard, Isaac Amankwaa, Patrick Broman, Marrin Haggie, Carlee Logan, Amy Pearce, Kesava Sampath, Ann-Rong Yan, Patrea Andersen

**Affiliations:** ^1^School of Health Sciences, Swinburne University, Hawthorn, QLD, Australia; ^2^School of Medicine and Dentistry, Griffith University, Gold Coast, QLD, Australia; ^3^Centre for Health and Social Practice and Centre for Sports Science and Human Performance, Waikato Institute of Technology – Te Pukenga, Hamilton, New Zealand; ^4^School of Nursing, Eastern Institute of Technology – Te Pukenga, Hawkes Bay, New Zealand; ^5^School of Nursing, Paramedicine and Healthcare Sciences, Charles Sturt University, Bathurst, NSW, Australia; ^6^School of Nursing and Midwifery, The University of Newcastle Central Coast Clinical School, Ourimbah, NSW, Australia; ^7^Faculty of Health, University of Canberra, Canberra, NSW, Australia; ^8^School of Nursing, Midwifery and Paramedicine, University of the Sunshine Coast, Sippy Downs, QLD, Australia; ^9^School of Nursing, Midwifery and Social Science, Central Queensland University, Sippy Downs, QLD, Australia

**Keywords:** interdisciplinary education, interdisciplinary communication, interprofessional relations, public health, primary healthcare, collaboration, assessment, measurement

## Abstract

Increasingly, interprofessional teamwork is required for the effective delivery of public health services in primary healthcare settings. Interprofessional competencies should therefore be incorporated within all health and social service education programs. Educational innovation in the development of student-led clinics (SLC) provides a unique opportunity to assess and develop such competencies. However, a suitable assessment tool is needed to appropriately assess student progression and the successful acquisition of competencies. This study adopts an integrative review methodology to locate and review existing tools utilized by teaching faculty in the assessment of interprofessional competencies in pre-licensure healthcare students. A limited number of suitable assessment tools have been reported in the literature, as highlighted by the small number of studies included. Findings identify use of existing scales such as the Interprofessional Socialization and Valuing Scale (ISVS) and the McMaster Ottawa Scale with Team Observed Structured Clinical Encounter (TOSCE) tools plus a range of other approaches, including qualitative interviews and escape rooms. Further research and consensus are needed for the development of teaching and assessment tools appropriate for healthcare students. This is particularly important in the context of interprofessional, community-partnered public health and primary healthcare SLC learning but will be of relevance to health students in a broad range of clinical learning contexts.

## 1. Introduction

Effective interprofessional engagement and collaborative practice are crucial to quality public health and primary healthcare delivery, especially given the growing prevalence of non-communicable illness (1). Therefore, fundamental skills of professional teamwork are essential to the preparation of 21^st^-century health and social workforces (2–5). Despite the necessity of pre-licensure healthcare students developing these interprofessional competencies, the educational experience and assessment process is often constrained by profession-specific boundaries and logistical barriers which require specific strategies to address (5–7). There is significant agreement that more work is needed in transforming curricula and effectively assessing the development of interprofessional competencies throughout the educational experience (8). This requires, for educators, the identification of interprofessional competencies required of members of healthcare teams and careful consideration of how these are taught and assessed (9). Prompted by the development of a student-led clinic in Aotearoa New Zealand, this search inquiry was undertaken to identify tools used globally by faculty to evaluate and assess interprofessional competencies in pre-licensure students from two or more healthcare professions. The search sought examples where two or more professions had worked together rather than tools developed or utilized from the activity and perspective of one profession alone.

## 2. Background

### 2.1. Student-led clinics

Student-led clinics (SLCs) are an increasingly widely used model of clinical practice education that increases the involvement of pre-licensure students in hands-on practice, particularly within primary healthcare settings, while providing a broad range of benefits to service users and communities (10). Of particular note, SLCs are shown to be a helpful health delivery model in providing public health and primary healthcare services to support underserved and marginalized health communities (1, 11, 12). SLCs may involve a single professional group (10) or may be interprofessional in nature (13, 14). The success of SLCs clinics is enhanced by thoroughly planning clinical activities, student experience and competency assessment. Detailed planning is vital if the clinics are interprofessional. While the benefits of interprofessional practice are well-understood, the IPE dimension adds more complexity to the endeavor of establishing an SLC (5, 6). Evidence-based pedagogical approaches are needed to inform the development of clinical placement rotations and experience.

### 2.2. Context

The researchers undertaking this review are involved in establishing an interprofessional SLC in the Waikato region of Aotearoa New Zealand. The region's high prevalence of non-communicable diseases such as Type two Diabetes Mellitus (T2DM), cardiovascular disease and respiratory illness calls for greater public health awareness and literacy and enhanced primary healthcare (15). An initial feasibility study canvassed the views of community organizations and members, enabling the proposed development to be community-led and aligned with the specific needs of local communities (16). Following community prioritization of need, it was agreed that the proposed SLC would focus on increasing public health awareness and enhancing primary healthcare access for a broad range of services related to T2DM and related non-communicable diseases. Services are intended to improve health knowledge and care access. Interprofessional delivery helps to address related equity issues (17). This integrative review was designed as part of the planning process for the SLC, to identify competency assessment tool/s currently being used by teaching faculty to inform the development of a teaching and assessment tool common to all pre-licensure students participating in the proposed SLC. Relevant professional groups include nursing, midwifery, physiotherapy, osteopathy, social work, counseling, clinical exercise physiology, dietetics, osteopathy, and sport science students.

### 2.3. Operational definitions

Ambiguity is not uncommon as various nomenclature is used within the literature to describe concepts of interdisciplinarity and assessment. Thus, definitions were explored as a precursor to this review with the following utilized for the purposes of the review.

#### 2.3.1. Interdisciplinarity

Interprofessional (IP), interdisciplinary and multidisciplinary practices are inconsistently defined in the literature. IP practice is perhaps best defined as multiple health team members from different professional backgrounds working together in clinical practice (18). In contrast, interdisciplinary practice involves “knowledge sharing” (19) from multiple knowledge bases and collaborating to achieve a shared outcome, typically with an educational focus (20, 21). Multidisciplinary practice is differentiated further, as professionals achieve this by working from their own knowledge base, with minimal/no knowledge of each other's knowledge base (19). IP is often also suffixed with education and learning. While IP practice refers to the clinical practice context, IP education and learning “occurs when two or more professions learn about, from and with each other to enable effective collaboration and improve health outcomes” (18) and is the process of preparing people for collaborative IP practice (22). Another important distinction to make is collaborative practice, when members of the healthcare teamwork with people from within their profession, people outside their profession, and multiple other stakeholders, such as patients/clients and their families or non-health members of the team (23). In this review, the focus is on assessment of IP practice in a clinical setting and, while this is an interdisciplinary context where collaborative practice will occur, the term IP will be used throughout.

#### 2.3.2. Assessment

This review searched for and appraised appropriate “tools” and “instruments” to inform how to best evaluate or assess IP practice in learners. Assessment “tools” and “instruments” are terms also used interchangeably in the literature (24–26), with contradictory definitions positioning assessment instruments as a component of assessment tools and vice versa (27, 28). For this review, the terms are interchangeable, and both are included as search terms, however, the term assessment tool is reported for consistency.

### 2.4. Research question

Our interests lie in understanding how competency for interprofessional practice has been measured, by teaching faculty, among pre-licensure healthcare students in practice settings (as opposed to the assessment of profession-specific competencies). Specifically, we sought to identify existing assessment tools used by faculty to assess interprofessional competency attainment of pre-li from two or more professions censure healthcare students in clinical learning contexts and which could be utilized within an interprofessional student-assisted clinic. Thus, this review focused on the following questions:

- What tools have been used by teaching faculty to assess interprofessional competencies of pre-licensure healthcare students experiencing learning in interprofessional contexts (i.e., involving two or more professions)?
- How might identified tools be used to inform development of an assessment instrument for assessing interprofessional competency attainment of healthcare students in clinical learning contexts such as a primary healthcare-focused interprofessional student-led clinic?

## 3. Method

This review was conducted using an integrative approach as described by Whittemore and Knafl (29). Interprofessional concepts and their associated measurement are complex and context specific (29). One study type or design cannot capture all the dimensions of healthcare students' interprofessional competency assessment and related tools. An integrative review allows for synthesizing methodologically diverse studies to comprehensively understand a particular issue or phenomenon to inform practice or policy (30). Adopting this methodology enables going beyond the narrow focus of traditional systematic reviews to ask broader, practice-based questions that can direct practice-based scientific knowledge (31, 32). The five integrative review methodology stages described by Whittemore and Knafl (31) – (1) problem identification, (2) literature search, (3) data evaluation, (4) data analysis, and (5) presentation – were utilized in this review.

### 3.1. Inclusion and exclusion criteria

The review's concepts and search terms were based on the PICO/PECO frameworks (**P**—Participants, **I/E**—Interventions/Exposure, C—Comparisons and **O**—Outcomes) (33). The selection criteria are summarized in Table 1. We placed no time restrictions; however, we included only studies published in English. The review includes primary studies only, excluding reviews, books, editorials, letters, and commentaries. Both qualitative, quantitative, and mixed methods studies were included.

**Table 1:** Inclusion and exclusion criteria.

	**Inclusion and exclusion criteria**	
Inclusion criteria	Population	Pre-licensure healthcare students at any level of study
	Intervention/exposure	interprofessional education and assessment
	Comparison	uni-professional education and assessment
	Outcome	**Primary**- interprofessional competency
Exclusion criteria	1.	Registered health professionals
	2.	Self-assessment of interprofessional competencies

### 3.2. Databases and search terms

We searched published materials and gray literature using three broad concepts (healthcare student, assessment and interprofessional competence) derived from our research question and refined by MeSH terms in Medline. An initial test string was tested in ERIC for relevance: (Pre-registration OR Pre-licensure) AND (Healthcare student OR Healthcare student) AND (postgraduate OR undergraduate) AND (Evaluate OR Assessment OR assessing OR assess OR outcome OR outcomes OR examin^*^ OR evaluate) OR (measurement OR measure OR measuring) AND (Competenc^*^ OR Competent) AND interprofession^*^) AND tools). We continued to develop this initial search strategy iteratively and tailor it across these databases: CINAHL, PubMed/Medline, Embase, ERIC and Proquest One Academic. Comprehensiveness in the search scope was achieved through a review of the reference list of relevant primary papers and other sources like Google and Google Scholar search. The search strategy is shown in Table 2.

**Table 2:** Search strategy on Proquest ONE Academic, ERIC, Medline and Embase, and search results on 25/05/2022.

Proquest ONE academic		
1	(Pre-registration OR Pre-licensure) AND (Healthcare student OR Healthcare student) AND (postgraduate OR undergraduate) AND stype.exact (“Scholarly Journals”) AND (measurement OR measure OR measuring AND tool^*^ OR scale) AND (Evaluate OR Assessment OR assessing OR assess OR outcome OR outcomes OR examin^*^ OR evaluate) AND stype.exact (“Scholarly Journals”) AND interprofessional	613
**ERIC**		
1	(Pre-registration OR Pre-licensure) AND (Healthcare student OR Healthcare student) AND (postgraduate OR undergraduate)) AND ((Evaluate OR Assessment OR assessing OR assess OR outcome OR outcomes OR examin^*^ OR evaluate) OR (measurement OR measure OR measuring) AND (Competenc^*^ OR Competent) AND interprofession^*^) AND tools) AND stype.exact(“Scholarly Journals”)	1867
**Medline (Via PubMed)**		
1	Healthcare Student [MeSH Major Topic]	30,385
2	Pre-registration OR Pre-licensure OR Postgraduate OR undergraduate	158,162
3	#1 OR #2	181,183
4	Assessment [Title/Abstract] OR Evaluate [Title/Abstract] OR Evaluation [Title/Abstract] OR Assessing [Title/Abstract] OR Assess [Title/Abstract] OR Outcome^*^[Title/Abstract] OR Examin^*^[Title/Abstract] OR Measurement [Title/Abstract] OR measure [Title/Abstract] OR measuring [Title/Abstract]	8,752,126
5	Competenc^*^[MeSH Major Topic]	2,670
6	Competenc^*^[Title/Abstract]	100,068
7	#5 OR #6	101,015
8	Interprofession^*^[Title/Abstract] OR Inter-profession^*^[Title/Abstract] OR Health profession^*^[Title/Abstract] OR healthcare profession^*^[Title/Abstract] OR Health [Title/Abstract] AND social care profession^*^[Title/Abstract] OR collaborat^*^[Title/Abstract]	179,031
9	#7 AND #8	5,199
10	#3 AND #4 AND #9	622
**Embase**<**1947 to present**>		
1	health student/	1686
2	(Pre-registration or Pre-licensure or Postgraduate or undergraduate).mp. [mp=title, abstract, heading word, drug trade name, original title, device manufacturer, drug manufacturer, device trade name, keyword heading word, floating subheading word, candidate term word]	93448
3	1 or 2	94751
4	((assessment or evaluation) and interprofessional).mp. [mp=title, abstract, heading word, drug trade name, original title, device manufacturer, drug manufacturer, device trade name, keyword heading word, floating subheading word, candidate term word]	5918
5	3 and 4	696
6	Competence/or clinical competence/	92987
7	5 and 6	92

### 3.3. Data screening and selection

**Figure 1:**
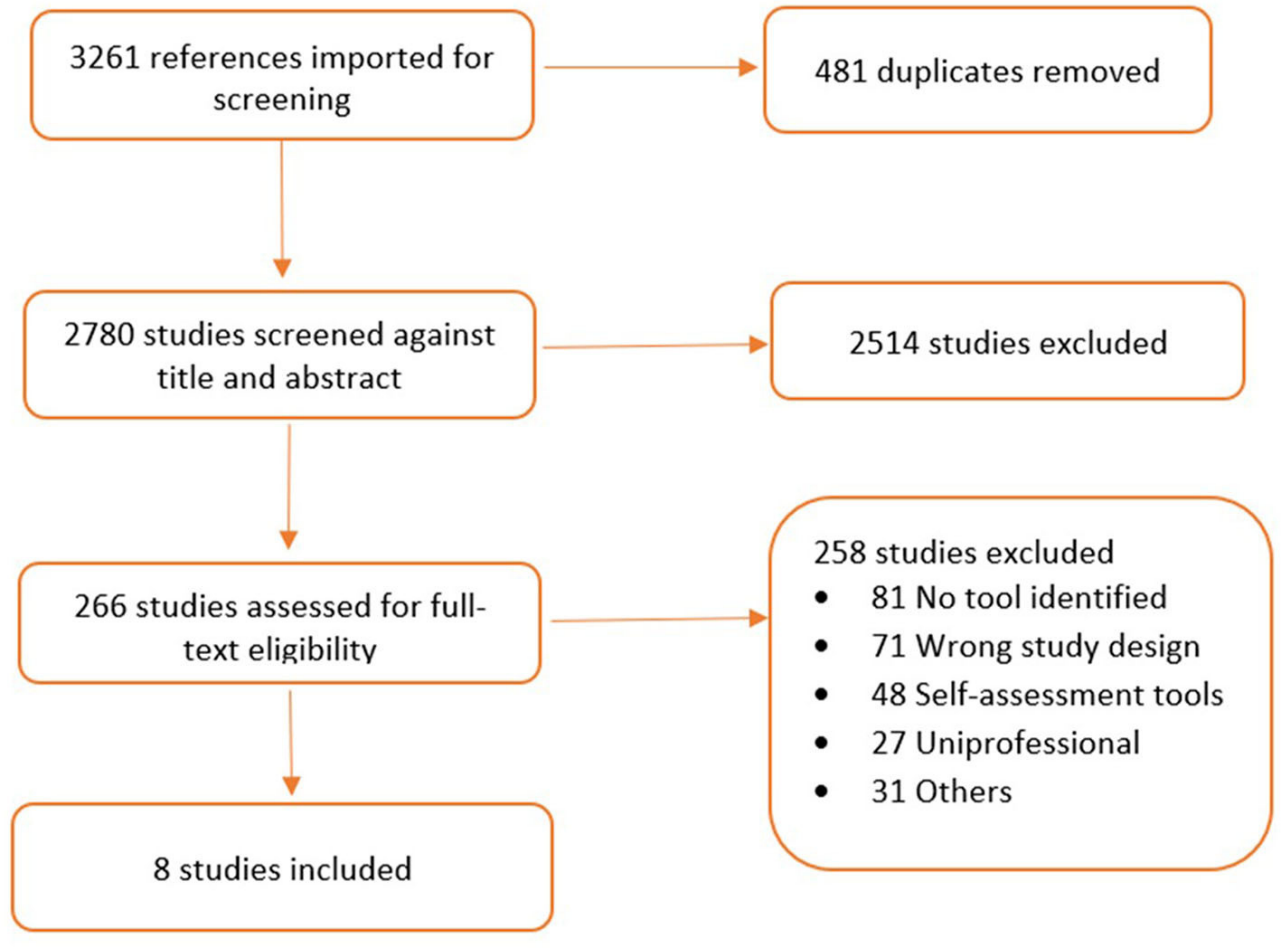
PRISMA flow chart of study selection process.

Identified records from databases and Google searches were imported into Covidence^®^ (34), an online screening and data management software. Automatic removal of duplicates in Covidence was followed by a two-staged screening of unique studies by two sets of independent reviewers including PB, SB, KKS, and IA. The initial screening of the titles and abstracts was followed by a further screening of full-text articles identified. Finally, a third and fourth reviewer (DB and A-RY) consulted together to resolve discrepancies and conflicts between the reviewer judgements in each stage of the review process. The screening and conflict resolution process in Covidence were blinded. The search strategy and data screening procedures, using the Preferred Reporting Items for Systematic Reviews and Meta-Analyses Protocols (PRISMA-P) Statements (35), are reported in Table 2 and Figure 1, respectively.

### 3.4. Data extraction and synthesis

Data were extracted and synthesized following Whittemore and Knafl (31) guidelines. The data extraction process involved reviewing each study's details, research design, aims, ethical considerations, sample population and size, comparative interventions, outcome measures, findings, and limitations. Covidence was used as the primary tool for data extraction. Data were then synthesized by identifying themes and concepts related to the review questions. The synthesis process involved sorting the data into intellectual bins, naming themes, and looking for relationships to guide future studies. The studies' psychometric features, such as internal consistency, inter-item and inter-total correlations, and inter-rater reliability, were examined to assess the quality and reliability of the findings. The key themes and relationships are summarized in **Table 5**.

### 3.5. Evaluation of data

Including both primary and theoretical literature in integrative review makes quality appraisal more complex (31). In line with our decision to integrate quantitative, qualitative and mixed methods studies, we adopted the “mixed-methods assessment tool (MMAT), version 2018” (36) for the quality appraisal of eligible studies. Two reviewers (DB and A-RY) independently appraised the quality of included studies and resolved any disagreements by consensus. Each study's quality is presented. In keeping with the integrative review methods, no eligible study was excluded based on research quality issues (31, 37).

## 4. Results

Eight manuscripts were identified for inclusion in the review (38–45), however, two reported activities from the same context. The PRISMA Flow Chart and study selection process (Figure 1) outlines the process of assessment and inclusion.

**Figure 2:**
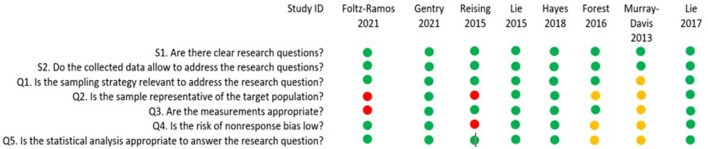
Quality appraisal of the included articles.

Application of the the ‘MMAT version 2018′ (36) provided the quality appraisal results shown in Figure 2.

In terms of study quality, notable issues exist where, sample representativeness is questionable due to the sample size being too small (38–40) or reported inconsistently (41). Selection bias may exist when the participants are recruited on a voluntary basis and if not all the participants are included for analysis (42). Also, the measurements may be inappropriate if only one rater is used in the competency assessment (41), and to assess tool quality. Bias is reduced when two faculty members rate and compare results vs. the assessment of a single faculty member alone.

Six of the eight studies were based in the United States of America, one in Canada and one in an unstated country. Each included diverse aims, as shown in Table 3. The different approaches included emphasis on the development and delivery of the interprofessional education program with the application of assessment tools (40, 41), or alternatively focusing on testing the assessment tools (38, 39, 42).

**Table 3:** Characteristics of the included studies.

**Study ID**	**Country**	**Aim of study**	**Limitations**	**Study design**	**Total number of students/assessors**
Foltz-Ramos et al. (41)	USA	To create and test the use of an interprofessional escape room to improve teamwork before interprofessional simulation	Previous experience of escape rooms was not considered; simulations rather than true life cases were used.	Quantitative descriptive studies	233/1
Gentry et al. (40)	USA	To describe a longitudinal, collaborative interinstitutional IPE project that engages community partners (CP) while delivering core IPE competencies.	Small sample size without medical students' participation; missing sociodemographic faculty data; not linking the student team to faculty assessment data; community-based IPE may be difficult to scale.	Quantitative descriptive studies	27/9
Reising et al. (42)	USA	To establish psychometric testing of the Indiana University Simulation Integration Rubric (IUSIR), a tool for measuring interprofessional communication in simulations	Agreement on how to score with the tool is needed when more than one behavior is involved; the sample consisted of nursing and medical student only from a single midwestern university; the tool is specific to individual and team communication; Simulation was used	Quantitative descriptive studies	295/NA
Lie et al. (35)	USA	To test the feasibility of using a retooled scale to rate performance in a standardized patient encounter and to assess faculty's ability to accurately rate both individual students and teams	Participants were trained students and one-third were lowest performing, which is not seen in real world; small sample size	Quantitative descriptive studies	16/16
Hayes et al. (43)	USA	To describe the IPE experiences and the development of Interprofessional Team-based Care Rubric (ITCR) and report its reliability and validity	A small size of sample from one regional university; the documentation was not graded; participants were at different academic levels; a nominal scale of zero to five rather than a more continuous scale was used	Quantitative descriptive studies	78/6
Forest et al. (44)	NA	to develop and implement a tool for rating teams and individuals	One institutional project; too few faculty trained to assess interobserver reliability statistically; the effect of giving feedback to the team was not investigated	Quantitative descriptive studies	NA/NA
Murray-Davis et al. (45)	Canada	To report on the development of a TOSCE for learners from three health professions from family physicians, midwives, and obstetricians	Next steps including assessor training and learner involved TOSCE are required	Quantitative descriptive studies	NA/NA
Lie et al. (39)	USA	To improve scale usability for clinical settings by reducing item numbers while maintaining generalizability; and to explore the minimum number of observed cases required to achieve modest generalizability for giving feedback.	A standard patient setting was used; only four health professions (Physician Assistant, Pharmacy, Occupational Therapy, and Nursing) were participated	Quantitative descriptive studies	63/16

The interprofessional initiatives assessed in the eight studies were equally diverse and included ongoing interprofessional activities; interprofessional collaboration with community partners; an interprofessional escape room; an interprofessional team-based care rubric, and a Team Observed Structured Clinical Encounter (TOSCE) station focused on stroke (see Table 4).

**Table 4:** Characteristics of the interprofessional education delivered in the included studies.

**Study ID**	**Name of IPP/IPE**	**Duration of IPP/IPE**	**Venue**	**Cases/patients**	**Participants**	**Raters**
Foltz-Ramos et al. (41)	Interprofessional escape room	NA	In a simulation center located in an eastern U.S. university	High-fidelity patient simulators	Third-year pharmacy and senior nursing students scheduled for an existing required session during the fall 2018 semester. An interprofessional simulation experience was part of mandatory coursework in their respective programs. Teams of four students: two pharmacy students and two nursing students	One observer
Gentry et al. (40)	MVA IPE collaborating with CP	six months 30 hours over two semesters	In a community setting	Na	Twenty-seven students from five universities representing ten healthcare academic programs were divided into five teams.	Nine faculty leaders
Reising et al. (42)	Ongoing interprofessional activities	At least one team simulation activity was planned per semester, with a minimum of four simulation activities for each student team throughout the curriculum	Na	Simulation scenarios	Two hundred and twenty nine pre-licensure bachelor of science in nursing students and 66 pre-licensures first- and second-year medical students. Teams consisted of one medical student and one to two nursing students	The lead nursing school faculty member and lead medical school faculty member
Lie et al. (35)	TOSCE station	35 minutes for one TOSCE station (stroke)	At the health science campus of a single institution (the University of Southern California) located in urban Los Angeles, California	Four sps were recruited from a database of experienced SP actors to perform at TOSCE stations with the selected case of stroke	Sixteen students from four professions were trained a priori to perform in teams of four at three different levels as individuals and groups	Sixteen volunteer faculty members, representing dentistry, medicine, occupational therapy, pharmacy, and physician assistant professions with experience teaching and assessing students and no prior experience with IPE assessment. Faculty members had a 60-minute pre-TOSCE training and were blinded to the study's purpose and student and IPE team performance levels
Hayes et al. (43)	NA	Phase I (Fall 2012 and 2013) began as one 3-hour experience with nursing and physical therapy students and faculty. Phase II started in Fall2014 and included two experiences during the semester and the addition of social work students and faculty.	At a regional comprehensive university in the southeast United States	The simulation scenario was based on an unfolding case study that followed one client from an acute care hospital admission through transitional care planning. Documentation assignments during the IPE experiences	Twenty five nursing students, 32 physical therapy students, 21 social work students. Students from the three programs were randomly assigned to ten teams of 7–8 students. Each team consisted of 2–3 nursing, 2–3 physical therapy, and 1–2 social work students.	Three raters and three additional raters
Forest et al. (44)	Training session	45 min	Na	Actor patient	Actors	40 faculty members
Murray-Davis et al. (45)	TOSCE stations	20 min	At an Ontario University who are involved in primary care obstetrics	A written description of a patient case, or a standardized patient, or a video monolog from a patient	Three professions	Two evaluators
Lie et al. (39)	A two-station TOSCE	Each station lasted 25 minutes	At the University of Southern California	Two standardized patients in succession	Sixty three volunteer students from the four health professions programs (Physician Assistant, Pharmacy, Occupational Therapy, and Nursing) no inclusion/exclusion criteria.	Sixteen volunteer faculty raters from the same four professions. The criterion was previous experience evaluating students in clinical settings. Review a standardized training video and complete the rating on the actor students, and a one-hour of in-person group training.

A single study (40) reported a multi-site inquiry of five sites; other studies involved single-site initiatives and evaluations. One study included four participating professions, namely, occupational therapy, pharmacy, dentistry and medicine (45) with the remaining studies involving fewer professions, for example, nursing and medicine (42) or nursing and pharmacy (41).

Each research team described their interprofessional assessment tool in detail and evaluated the performance in their specific study context (see Table 5) Five assessment tools were used across the 8 studies, none of which are the same, though four of them are modified from the McMaster-Ottawa scale in different ways (38–40, 52) Two studies evaluated internal consistency of the assessment tools (Observed Interprofessional Collaboration [OIPC] and Indiana University Simulation Integration Rubric [IUSIR], respectively) and reported the Cronbach's alphas, which ranged from 0.79 to 0.91 indicating a high reliability (38). Two studies analyzed interrater reliability of the assessment tools (IUSIR and TOSCE) between two and sixteen assessors, respectively (38, 42): Reising et al. reported high accuracy for both individual (92%) and team (94%) assessment by IUSIR from two assessors, while Lie et al. found a lower accuracy in individual (38–81%) than team (50–100%) assessment by TOSCE from sixteen faculty raters. These two studies also validated the assessment tools. The assessment tool IUSIR was found to have significant discriminatory capacity to differentiate junior-/senior-level performance (42); however, with the assessment tool TOSCE individual but not team performance may be over-rated (38).

**Table 5:** Characteristics and performance of the assessment tools applied in the included studies.

**Study ID**	**Name of the tool**	**Outcome measured**	**Items**	**Scales**	**Internal consistency**	**Inter-item & inter-total correlations**	**Interrater reliability**	**Scores/validity**	**Themes**	**Summary**
Foltz-Ramos et al. (41)	Observed Interprofessional Collaboration (OIPC)	Interprofessional collaboration	The first ten items relate to the adequacy of how the team builds a shared vision of the situation and the remaining ten items relate to the team's ability to develop a joint action plan.	For each item, teams are rated using a 3-point Likert-type scale (1 = inadequate, 2 = more or less adequate, 3 = adequate).	The Cronbach alpha was: 0.84 for the first ten items on the OPIC; 0.82 for the remaining ten items on the OIPIC; and 0.91 for the overall score indicating high reliability for each	NA	NA	Total score: control group 53 (43, 44, 46–51) vs. intervention group 55 (43, 49–51), *p* < 0.01 Items 1–10 Subtotal score: control group 26 (24–28) vs intervention group 27 (26–28), *p* < 0.01 Items 11-20 Subtotal score: control group 27 (25–28) vs. intervention group 27 (26–28), *p < * 0.01	Enhanced teamwork	Participating in escape rooms improved teamwork and performance during simulation, as measured by the OIPC and ISVS-21 instruments. The intervention group, which participated in the escape room activity, had higher median scores in team building, common action plan development, and overall total score compared to the control group. The control group, on the other hand, had more students who were able to escape the escape room, and those who did not escape needed more suggestions than those who did. While the escape room activity does not increase individual problem-solving skills, it does improve teamwork and collaboration among students in an interprofessional education context
Gentry et al. (40)	five-item modified TOSCE Scale	Interprofessional team competency	1. Collaboration 2. roles and responsibilities 3. community partner centered approach 4. conflict management and resolution 5. values and ethics	per item: 3 (minimum)−9 (maximum) points total score: maximum 45 points	NA	NA	NA	Average total score: 43.11 (+/- 3.26) Average scores per item: collaboration 8.67 (+/- 0.71), roles and responsibilities 8.56 (+/- 1.01), community partner centered approach 8.67 (+/- 0.71), conflict management and resolution 8.67 (+/- 0.71), values and ethics 8.56 (+/- 1.01)	Interprofessional Education (IPE) and Enhanced Teamwork	Most students expressed interest in Interprofessional Education (IPE) and collaboration for future collaborations. A follow-up assessment with 21 students showed significant changes in attitudes, behaviors, and beliefs about interprofessional collaboration and socializing. ISVS total scores also significantly improved, with collaboration, communication, and comfort with other professions being recurrent themes. Faculty leaders assessed program student teams using a modified Team Objective Structured Clinical Examination (TOSCE) Scale, which resulted in high scores in collaboration, responsibilities, tasks, community partner-centered approach, conflict management and resolution, values, and ethics
Reising et al. (42)	Indiana University Simulation Integration Rubric (IUSIR)	interprofessional communications	Individual Body language, Eye contact, (Physical) Appearance; Use of closed-loop communication, Use of terminology, Introduction to the patient; Incorporating feedback, Asking for clarifications and questions, Addressing errors; Seeking out input from the team, Referring to written resources; Identifying critical patient care issues, Implementing treatment; Patient reassurance, Addressing patient questions. Team Teams' energy and communication; Using closed-loop communication; Using input, Patients' care; Clinical impression; Education of patient about treatment; Reassessing patient after treatment.	For each item, the lowest performing score is 1, the mid-score is 3, and the high score is 5. The maximum score for an individual and a team is 30.	The Cronbach's alphas for individual items: nursing students 0.82 medical students 0.86 The Cronbach's alphas for team items: nursing students 0.79 medical students 0.90	The average individual inter-item correlation was 0.434; the average team inter-item correlation was 0.3906 The average individual inter-total correlation was 0.517; the average team inter-total correlation was 0.479	for individual scores 92% for team scores 94%	For nursing scores on individual items, senior-level students performed significantly better than junior-level students, *p* < 0.000. Senior-level team scores on team items were significantly higher than junior-level team scores, *p* < 0.001	Communication Skills Assessment	IUSIR is a reliable and valid tool for measuring individual and team communication skills in simulated environments; Senior-level students outperformed junior-level students on individual and team items; Overall, the IUSIR is a useful tool for measuring interprofessional communication skills in simulated environmen
Lie et al. (35)	TOSCE modified from the McMaster-Ottawa scale	Interprofessional individual and team competencies	Rating individual students: 1. Communication Assertive communication Respectful communication Effective communication 2. Collaboration Establishes collaborative relationships Integration of perspectives Ensures shared information 3. Roles and responsibilities Describe roles and responsibilities Shares knowledge with others; accepts accountability 4. Collaborative patient-family-centered approach Seeks input from patients and family Shares with patients and family Advocates for patient and family 5. Conflict management/ resolution Demonstrates active listening Respectful of different perspectives Works with others to prevent conflict 6. Team functioning Evaluates team function and dynamics Contributes effectively Demonstrates shared leadership	1 or 2 or 3 point for each item	NA	NA	Accuracy of faculty raters: 38-81% of individuals, 50-100% teams.	with errors in the direction of over-rating individual, but not team performance	Faculty evaluation	Faculty demonstrated a leniency error in rating students, even with prior training using behavioral anchors; Two trained faculty raters per station are recommended to improve consistency; G-study shows most of the variance in student scores was attributable to systematic differences between students; Faculty expressed a need for more training and a simpler rating form
Hayes et al. (43)	Interprofessional Team-based Care Rubric (ITCR)	student team learning	ITCR tool is comprised of five major items, each of which contains five key criteria for a total of 25 key criteria. The Interprofessional Collaborative Practice Competency Domains from IPEC were used to inform the criteria standards, which are (1) values/ethics for interprofessional practice, (2) roles/responsibilities, (3) interprofessional communication, and (4) teams and teamwork	1 not relevant, 2 somewhat relevant, 3 quite relevant, 4 highly relevant The total team scores were reported as an average of 5 instead of a total of 25	NA	NA	The ITCR was found to have good reliability in testing (0.842) by 3 raters who used the rubric to evaluate student performance on a sample of 30 team documentation assignments during the development process, and (0.825) for all rubrics by three additional raters	For the five major items of the ITCR, both the item-level and scale-level content validity index (CVIs) were 1.00, indicating the scale was determined to have excellent content validity. For the25 key criteria, the item-level CVI has a range of 0.67e1.00. Three criteria did not achieve universal agreement among the raters. The scale-level CVI was 0.96, which is above 0.90 and considered acceptable	Rubric Development and Assessment	The rubric building process revealed that the three professions have different vocabulary and professional boundaries. The Interprofessional Team Communication Rubric (ITCR) data demonstrated statistical variations in team performance between labs, with lab 1 having the highest performance and lab 3 the lowest. However, teams performed similarly across the three labs and the rubric was found to be useful in detecting performance discrepancies and guiding team development. The tiny sample size limits the study, but it emphasizes the difficulty of creating a uniform interprofessional assessment tool and highlights the need for continual evaluation of interprofessional education experiences
Forest et al. (44)	modified McMaster-Ottawa Scale	student and interprofessional team performance	Six competencies are communication, collaboration, roles and responsibilities, collaborative patient-family centered approach, conflict management and resolution, teamwork/team functioning, and global score.	3 points scale: 1 below expected 2 at expected 3 above expected	NA	NA	NA	NA	Online and Hybrid Learning	There are three major themes that emerged: (1) the impact of technology on education, (2) the importance of student engagement and participation, and (3) the challenges and opportunities presented by online and hybrid learning. Within these themes, several patterns and relationships were identified, including the increased use of online learning tools, the need for personalized and interactive learning experiences, and the importance of effective communication and support for students in online and hybrid environments.
Murray-Davis et al. (45)	McMaster-Ottawa observer score based on the Canadian Interprofessional Health Collaborative's National Competency Framework	Collaborative Competency	communication, collaboration, roles/responsibilities, collaborative patient-family centered approach, conflict management/ resolution, and team function	NA	NA	NA	NA	NA	Communication Skills Assessment.	Internal consistency was supported for all individual and team items, and inter-item and inter-total correlations were positively correlated. Interrater reliability was also high. The tool was found to be a reliable and valid measure for interprofessional communication, with sensitivity to changes in communication skills over time. Senior-level students outperformed junior-level students on individual and team items. Overall, the IUSIR is a useful tool for measuring interprofessional communication skills in simulated environments.
Lie et al. (39)	Modified McMaster-Ottawa scale	interprofessional team competencies	Seven items: Collaboration, Roles, Patient/Family-centeredness, Conflict Management, Communication, Teamwork, and Global. Four items: Collaboration, Roles, Patient/Family-centeredness, and Conflict Management	3 points with descriptive behavioral anchors	NA	NA	NA	Team scores from a two-station TOSCE demonstrate low generalizability whether the scale consisted of four (0.53) or seven items (0.55) Individual scores from a two-station TOSCE demonstrate modest generalizability whether the scale consisted of four (0.73) or seven items (0.75)	Individual Performance Assessment	Observation of students in teams interacting with two different patients provides reasonably reliable ratings for giving feedback; Team scores from a two-station TOSCE demonstrate low generalizability whether the scale consisted of four or seven items

## 5. Discussion

The authoring team closely followed Whittemore and Knafl (31) five integration stages in conducting this review: (1) problem identification, (2) literature search, (3) data evaluation, (4) data analysis, and (5) presentation. During the first stage of the review the team clarified the need to seek, locate and review existing tools utilized by teaching faculty in the assessment of interprofessional competencies of relevance to pre-licensure healthcare students. The second through fourth stages of literature search, evaluation and analysis are reported in Sections 2.2 to 2.5 with results presented in Tables 3, 4. The final presentation of results is aided by the analysis in Table 5 and the ensuing discussion.

Results yielded a paucity of published work in the field. The search focused on identifying examples where faculty had worked together in the development and evaluation of IPE competency assessment tools for pre-licensure students from two or more healthcare professions. The identified tools included the OIPC, a five-item modified TOSCE Scale, the IUSIR, TOSCE modified from the McMaster-Ottawa scale, the Interprofessional Team-based Care Rubric (ITCR), the modified McMaster-Ottawa scale, and others.

The reported consequences of deficits in interprofessional communication and teamwork include increases in medical errors, poor patient outcomes and persistence of embedded health inequalities (17, 41). As early as the 1970's, entities such as the World Health Organization (WHO) and the Institute of Medicine (IOM) highlighted the need for an increased focus on public health and primary healthcare supported by increased collaboration between the professions (53, 54). The IOM Conference of 1972 focused specifically on the transformation of health professional curricula to address the increasingly important need for interprofessional education (53). The ensuing decades have seen continuing calls for curriculum transformation and emphasis on interprofessional education (3, 18, 46, 55, 56) and yet significant work remains to be done. A clear finding of this review is that while progress has been made, major gaps persist in various aspects of curriculum transformation, IPE pedagogy and assessment processes. Additional development and research are needed in respect to the education and assessment of interprofessional competencies among health professionals including pre-licensure healthcare students (5, 47).

Despite the small volume of work identified in this search, valuable insights were gained regarding assessment tools that could be utilized with pre-licensure healthcare students in an IP SLC service or other clinical learning context. Lie et al. (38) adopted an existing scale, specifically, the 9-point McMaster-Ottawa Scale and associated TOSCE tool (44, 48) and converted this to a 3-point scale with behavioral anchors. Participating faculty indicated comfort in assessing up to four students within the TOSCE period of 35 minutes. However, a leniency error was noted among faculty even after comprehensive training. It is recommended that two trained faculty raters are included in each TOSCE station (38). The McMaster-Ottawa Scale was also adapted by Forest et al. (44) to develop a three-point scale, with Lie et al. (39) building on their earlier developments – Forest and Lie both reported the usefulness and validity of the McMaster- Ottawa Scale as a basis for development and implementation (39, 43).

In the ITCR approach utilized by Hayes et al. (43), interprofessional practice competency domains were used to inform the criteria standards within the tool. Testing occurred in respect to both the level and content of the scale with results showing excellent content validity (49). Reising et al. (42) undertook psychometric testing using the IUSIR which is a tool that has been developed to measure interprofessional communication during clinical simulation (42). While useful, the tool is somewhat narrow in focus in that it assesses the interprofessional communication domain only rather than a broader set of interprofessional competencies. A further limitation is that design and testing using the IUSIR tool has occurred in simulated contexts only, with utility in practice contexts yet to be determined.

The use of an interprofessional escape room is reported by Foltz-Ramos et al. (41) to improve and test interprofessional collaboration in pre-license nursing and pharmacy students (41). Escape rooms are a relatively recent teaching innovation that integrates gaming technology with learning – an attractive approach among 21^st^-century learners (50). Escape room technology requires students to cooperate to effectively escape a particular scenario and achieve a good outcome. Escape rooms help build teamwork skills. The tool was shown to be effective, however, escape room development requires high levels of technical expertise and resource (41) and while fruitful they are essentially a simulated learning activity and further innovation is required to implement within the context of clinical rotations such as SLCs (41).

Transforming curricula to strengthen the focus on public health and primary healthcare priorities and reduce healthcare inequalities must take the student out of the classroom and into the community (51). However, studies reporting IPE assessment in the community and SLC settings are not commonly reported (40). Uniquely, Gentry et al. (40) collaborated with community partners over six months to deliver and assess interprofessional competencies of pre-licensure students in practice settings within primary care settings. Teams were drawn from ten professional groupings across five universities with a mixed-method approach taken to education and assessment. Participating community partners were not-for-profit entities delivering services to specific underserved and vulnerable populations. Faculty undertook continuous assessment and provided feedback to students throughout the six-month placement. Faculty assessments included qualitative assessment of IP domains; feedback on student presentation to community partners; utilization of existing tools specifically, the Interprofessional Socialization and Valuing Scale (ISVS) (57) completed prior to and after the placement; use of the McMaster-Ottawa Scale and TOSCE assessments, and analysis and feedback on student reflections.

The ISVS is a 24-point self-reporting measure focused on attitudes, behaviors and beliefs that underpin interprofessional socialization. The scale is used before and after the educational/clinical placement experience with a view to measuring the impact of the placement experience (57). The McMaster Ottawa Scale with TOSCE was explicitly developed for assessments of interprofessional competencies in primary care with the view to enable public health and primary healthcare teams to assess and then improve their team collaboration competencies – patient safety and better outcomes being a major aim (44, 48). In the Gentry et al. (40) study faculty utilized each of these assessment and feedback tools. Students reported a major benefit of the experience as getting to know the perspectives of others and working with like-mind people who also brought entirely different skill sets (40). Faculty and students also reported a greater understanding and comfort with team-based roles, improved competence in shared decision-making and problem-solving, and a greater understanding and empathy for community needs (40). The mixed method, community-based approach detailed by Gentry and team aligns well with a community-based, student-led interprofessional health service, the development of which formed the impetus of this search.

The identified tools provide valuable insight into the development of an assessment instrument for evaluating interprofessional competency attainment of healthcare students in clinical learning contexts, such as a primary healthcare focused interprofessional student-led clinic. While unvalidated, the McMaster-Ottawa Scale with TOSCE and the ISVS seem to show the greatest promise as tools for this purpose. The McMaster-Ottawa Scale with TOSCE is designed for assessing interprofessional competencies in primary care settings, enabling teams to evaluate and improve their collaborative skills, ultimately aiming for better patient safety and outcomes (38). The ISVS is a 24-point self-reporting measure that focuses on attitudes, behaviors, and beliefs underpinning interprofessional socialization (40, 51), which can be used before and after educational or placement experiences to gauge the impact of these experiences on students' interprofessional competency development.

When developing an assessment instrument for a primary healthcare focused interprofessional student-led clinic, it may be beneficial to incorporate elements from these existing tools while adapting them to the specific context and learning objectives of the clinic. Combining a mixed-method approach that includes continuous assessment, feedback loops, and strong community engagement, as demonstrated in the Gentry et al. (40) study, can further enhance competency development and assessment. Utilizing a variety of assessment methods such as self-reporting, qualitative assessments, and observed clinical encounters will provide a comprehensive evaluation of interprofessional competency development among students. Ultimately, ongoing research and evaluation are essential to refine any assessment instrument and ensuring its effectiveness in fostering interprofessional competencies in future healthcare professionals.

### 5.1. Limitations

It is appropriate to note some limitations of this review. Perhaps most obvious is the possibility that the search did not capture all relevant literature, especially given the heterogenous nature of terminology used to describe practice involving representatives from more than one health profession; and an assessment or measurement instrument. Determining what was a tool used by teaching faculty to assess (as opposed to self-assessment) was also difficult. Including only published articles in the English language may have excluded examples of international examples or tools in the gray literature, especially as teaching and learning tools are often informal and evolving and not always well-documented. Educators working to promote interprofessional collaboration among health profession students, and formally assessing the results, should be encouraged to share the tools or applications they have built or explored to do so. Additionally, each of the identified works was very different. The majority were based in the USA and one in Canada, where there is a strong emphasis on interprofessional practice collaboration across all health professional accrediting bodies (47). The lack of global representation in the identified studies is noted as a limitation within the findings of this review.

## 6. Conclusion

Effective interprofessional teamwork is a cornerstone to improved health outcomes and reductions in healthcare inequalities. Purposefully designed placement experiences and assessment activities are required to better develop interprofessional competencies among pre-licensure healthcare students and prepare them for practice. The mixed method assessment approach with continuous feedback loops and strong community engagement aligns well with the planning and delivery of a student-led clinic engaged delivering of public health and primary healthcare services. Existing assessment tools, such as the ISVS and the McMaster Ottawa Scale with TOSCE can further guide assessment processes and form the basis of future tool validation studies. Ongoing research and validation studies are needed to inform education and practice developments in this field of interprofessional competency assessment tools for faculty assessing students.

## Data availability statement

The raw data supporting the conclusions of this article will be made available by the authors, without undue reservation.

## Author contributions

SB, PA, and PB conceived the evaluative design of the study. SB, DB, A-RY, and IA developed the search strategy. All authors provided substantial contributions to this work and accept accountability for the finished product, participated in the collection of data, contributed to data analysis including COVIDENCE screening and writing of the manuscript, and reviewed and approved final drafts.
